# Cerebrospinal fluid metabolites and multiple sclerosis: A two-sample Mendelian randomization study

**DOI:** 10.1097/MD.0000000000046960

**Published:** 2026-01-02

**Authors:** Dejin Zhang, Jiaojiao Feng, Jing Feng, Gang Chen

**Affiliations:** a Department of Pharmacy, The People’s Hospital of Linshui, Guangan, China; b School of Pharmacy, Hebei North University, Zhangjiakou, China.

**Keywords:** causality, cerebrospinal fluid metabolites, genome-wide association study, Mendelian randomization, multiple sclerosis

## Abstract

Epidemiological evidence suggests that cerebrospinal fluid (CSF) metabolites are associated with multiple sclerosis (MS). However, the causality remains unclear as a consequence of confounding or reverse causation. As a result, we evaluated the causal relationship between CSF metabolites and MS. A 2-sample Mendelian randomization analysis was performed for 338 CSF metabolites and MS, with causal relationship elucidated based on genome-wide association studies (GWAS) data. Summary statistics for 338 CSF metabolites were retrieved from a GWAS of 689 participants, and summary statistics for MS consisted of GWAS data including 115,803 participants (47,429 patients and 68,374 controls). The inverse-variance weighted method has been applied to perform primary analysis, and extensive sensitivity analyses were completed to guarantee robustness. The inverse-variance weighted analysis revealed a suggestive causal correlation between higher beta-alanine (odds ratio [OR] = 1.248, 95% confidence interval [CI] 1.092–1.427, *P* = 1.16 × 10^-3^), 1-stearoyl-2-arachidonoyl-gpc (18:0/20:4) (OR = 1.090, 95% CI 1.026–1.159, *P* = 5.48 × 10^-3^), allantoin (OR = 1.123, 95% CI 1.021–1.236, *P* = 1.68 × 10^-2^), and heightened susceptibility of MS, an increased risk of MS. By comparison, Wald ratio analysis indicated that N,N,N-trimethyl-l-alanyl-l-proline betaine (OR = 0.934, 95% CI 0.894–0.976, *P* = 2.57 × 10^-3^), butyrate (4:0) (OR = 0.908, 95% CI 0.840–0.982, *P* = 1.61 × 10^-2^), and N-acetylglycine (OR = 0.909, 95% CI 0.836–0.987, *P* = 2.29 × 10^-2^) were significantly connected to a reduced MS risk. In addition, sensitivity analyses her eliminated potential biases arising from heterogeneity and horizontal pleiotropy. We present indicative genetic data that support a causal correlation between CSF metabolites and MS, pointing out the possibility of intervening CSF metabolites to prevent MS.

## 1. Introduction

Multiple sclerosis (MS) ranks among the most common chronic neurological disorders, mainly affecting young people and could result in disability. With a prevalence of 0.05% to 0.3%, MS exhibits significant regional differences on a global scale. Specifically, MS is particularly common in areas inhabited by Northern Europeans, temperate regions, and high-income countries, while MS is not common in areas inhabited by nonwhite population, low-income countries, and tropical regions.^[1]^ Currently, there is a fast-growing global trend in the occurrence and overall prevalence of MS,^[2–5]^ which would cause great burden to the society. The clinical manifestations of MS often include neurological dysfunction such as urinary and fecal incontinence, sensory loss, and difficulty in moving.^[6]^ Furthermore, MS is prone to recurrence, leading to functional loss and low life quality. Also, there is currently no clear cause for MS, bringing difficulty for clinical management^[7]^ and resulting in even more social and economic burden.^[8]^ However, difficulty in obtaining specimens and related risks have posed more significant challenges. Therefore, identifying biomarkers for MS is of great importance, which could potentially benefit many patients.

Cerebrospinal fluid (CSF) constitutes the primary extracellular fluid within the central nervous system. And analysis of CSF can better reflect the intrinsic changes and metabolic characteristics of the central nervous system. CSF offers an exceptional insight into the brain’s internal milieu, providing key insights into metabolic changes associated with neuropathology.^[9–11]^ Study by Cross et al^[12]^ have shown that elevated CSF glial fibrillary acid protein was associated with progression of MS. Additionally, some other CSF metabolites such as glycine, have demonstrated atypical alterations linked to MS.^[13]^ According to other findings,^[14]^ there was a significant connection of CSF l-glutamate levels and MS onset. More and more studies have suggested that CSF metabolites were associated with the onset of MS. However, the correlation between MS and elevated CSF metabolites are still unclear. Monitoring changes in CSF metabolite levels can further clarify their relationship with MS, providing opportunities for diagnosis and management.

Mendelian randomization (MR) analysis represents an epidemiological approach employed to deduce causal correlations between exposure and outcome based on genetic variation.^[15–17]^ MR analysis significantly mitigates the influence of confounding factors and reverse causality, since genetic variants are randomly allocated at conception.^[18–21]^ As a consequence, 2-sample MR method was utilized to examine the causal correlation of genetic predisposition to 338 CSF metabolites and MS risk.

## 2. Methods

### 2.1. Study design

**Figure 1.**
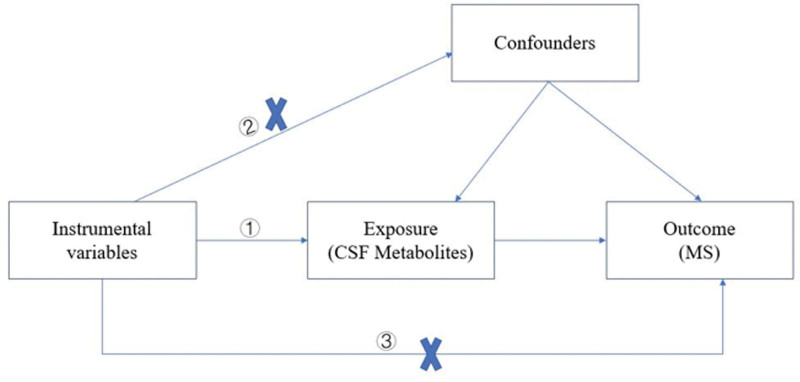
Basic assumptions of the MR study.

We conducted a 2-sample MR analysis to explore the causal correlation between 338 CSF metabolites and MS, with flowchart shown in Figure 1. The selection of single nucleotide polymorphisms (SNPs) as instrumental variables (IVs) is built upon the 3 principal assumptions of MR: IV should be closely related to exposure; IV must remain unassociated with any potential confounding factors; and IV was associated with outcome through exposure.^[19]^ In addition, strengthening the reporting of observational studies in epidemiology using Mendelian randomization checklist is displayed in Table S4, Supplemental Digital Content, https://links.lww.com/MD/R77.

### 2.2. Genetic instrument selection

In order to ensure validity, we conducted relevant screening of IVs from SNPs. Firstly, in this study, to guarantee the inclusion of sufficient IVs, SNP loci with *P* < 5 × 10^-5^ were selected for primary analysis.^[22]^ Secondly, to avoid the impact of strong chain imbalance, we set the threshold to *R*^2^ < 0.001, kb = 10000. Finally, the selected SNPs data were extracted from the outcome variables.^[23–26]^ In addition, to remove the impact of weak IVs, *F* test was utilized for evaluation and SNPs with *F* values <10 would be excluded.

### 2.3. Data sources

The genome-wide association studies (GWAS) data for 338 CSF metabolites were extracted from Wisconsin Alzheimer Disease Research Center (WADRC) and Wisconsin Alzheimer Disease Prevention Registry (WRAP) study. Our study comprised 689 individuals, including 532 from WADRC and 168 from WRAP, both of which were European population.^[27]^ Lumbar puncture was used to obtain CSF specimens, and identical collection and storage protocols were applied in both cohorts, consistent with earlier reports. GWAS summary statistics of MS patients were obtained for the primary analysis, including 47,429 patients and 68,374 controls of European ancestry.^[28]^ All studies incorporated in our analysis received approval from their respective ethics committees, and written informed consent was obtained from all participants in the original cohorts.

### 2.4. Statistical analysis

This study used random effects inverse variance-weighted (IVW),^[29,30]^ weighted median,^[31]^ MR Egger regression,^[32–34]^ and weighted models^[35]^ to verify the causal correlation between 338 CSF metabolites and MS. Among these approaches, IVW is commonly employed, and its results are considered relatively robust as it calculates the weighted mean of effect estimates derived from all IVs. As a consequence, we utilized results obtained from IVW as primary evaluation. In addition, weighted median, MR Egger, and weighted mode analysis were also performed to exclude potential confounding factors. To determine the robustness of the results, we conducted analyses on heterogeneity and horizontal pleiotropy tests, as well as Leave One Out test to examine sensitivity. To be more specific, MR-PRESSO was initially implemented to assess horizontal pleiotropy, removing detected outliners for subsequent MR reanalysis. Moreover, Cochran *Q* test would be applied, with *Q* value over 0.05 suggesting no heterogeneity. All the IVs satisfied the 3 main assumptions, making IVW method statistically effective. Furthermore, other 3 MR methods was conducted as supplements to minimize the existence of heterogeneity and horizontal pleiotropy. Throughout this study, Two Sample MR and MR-PRESSO packages were combined, with all analysis completed in R software (version 4.4.0; Medicine IT, Guangzhou, China). In order to avoid false positives due to multiple tests, the false discovery rate (FDR) correction was performed, and *P*_FDR_ < .05 was considered statistically significant. When *P* < .05 and *P*_FDR_ ≥ .05, CSF metabolites were considered to have a potential suggestive association with MS.

## 3. Results

### 3.1. Genetic instrument selection

This study followed strict IV selection criteria and ultimately selected GWAS data of 388 CSF metabolites as exposures, including IVs ranging from 14 to 144. All IVs underwent MR Steiger filtering to ensure the consistent removal of outliers from the finalized dataset. Moreover, the *F* statistic of all IVs were over 10, mitigating bias arising from weak instruments. Table S1, Supplemental Digital Content, https://links.lww.com/MD/R76 shows details on selected IVs with significant causal evidence. Additionally, specific information on all selected IVs and related results could be found in Tables S1 and S2, Supplemental Digital Content, https://links.lww.com/MD/R76.

### 3.2. Association of exposure and outcome

**Figure 2.**
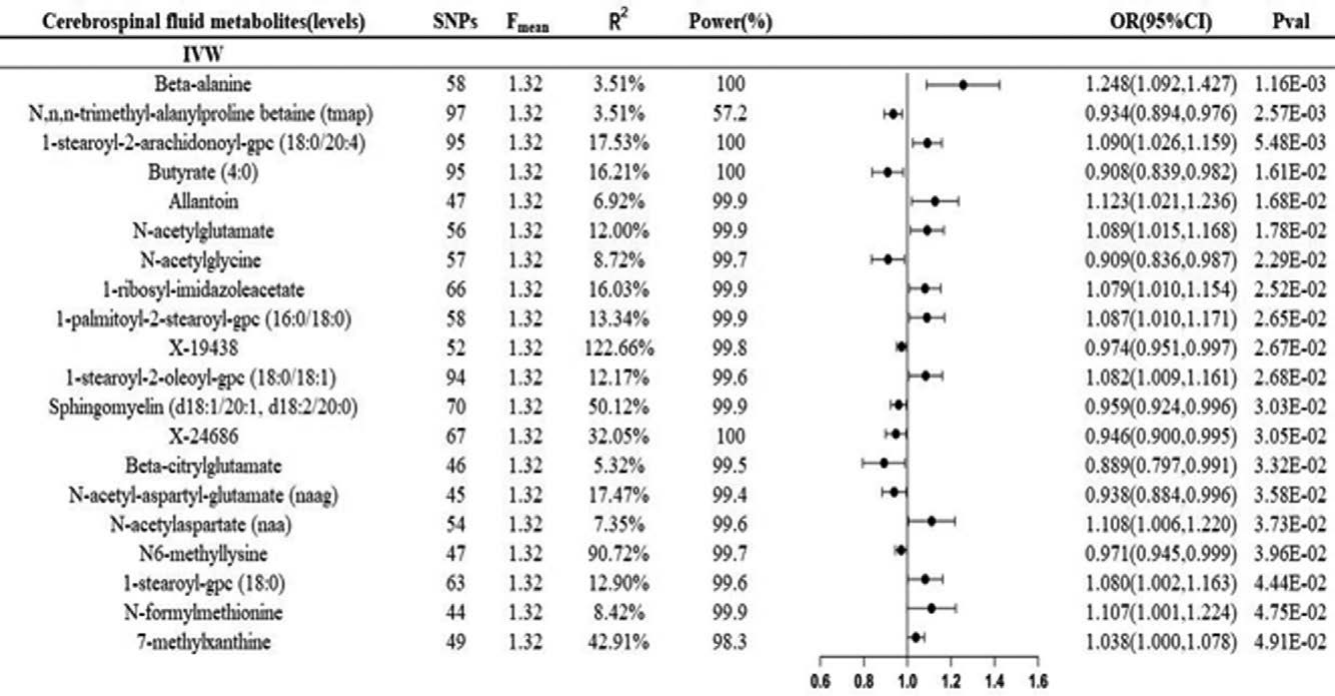
Summary of all significant IVM results with based on at least 2 SNPs. CI = confidence interval, IVW = inverse-variance-weighted, OR = odds ratio, SNP = single nucleotide polymorphism.

In the IVW method, 20 CSF metabolites were identified to be associated with MS (Fig. 2). Specifically, suggestive causal correlation was demonstrated between higher levels of beta-alanine (odds ratio [OR] = 1.248, 95% confidence interval [CI] 1.092–1.427, 1.16 × 10^-3^), 1-stearoyl-2-arachidonoyl-gpc (18:0/20:4) (OR = 1.090, 95% CI 1.026–1.159,*P* = 5.48 × 10^-3^), Allantoin (OR = 1.123, 95% CI 1.021–1.236, *P* = 1.68 × 10^-2^) and MS. Each standard deviation increase was connected to a 24.8%, 9%, and 12.3% heightened risk of MS, respectively. Conversely, Wald ratio analysis indicated that N,N,N-trimethyl-l-alanyl-l-proline betaine (TMAP) (OR = 0.934, 95% CI 0.894–0.976, *P* = 2.57 × 10^-3^), butyrate (4:0) (OR = 0.908, 95% CI 0.840–0.982, *P* = 1.61 × 10^-2^), and N-acetylglycine (OR = 0.909, 95% CI 0.836–0.987, *P* = 2.29 × 10^-2^) were suggestively associated with reduced risk of MS. Moreover, each standard deviation increase was related to lower MS risk of 6.6%, 9.2%, and 9.1%, respectively.

A range of sensitivity tests was carried out to confirm the robustness of the findings. The MR Egger generated 338 outcomes, all of which indicated no horizontal pleiotropy. Cochran *Q* statistic was applied to cases with multiple IVs, yielding 338 outcomes with no heterogeneity found (*P* > .05). In addition, the use of random effects models in these analyses mitigated potential biases caused by heterogeneity. MR-PRESSO analysis excluded all outliers and did not provide evidence of horizontal pleiotropy. Detailed results from sensitivity are presented in Table S3, Supplemental Digital Content, https://links.lww.com/MD/R76, without showing any causal relationship driven by single SNP.

According to our results, all IVs were strongly correlated with exposure. Therefore, while retaining these IVs, other methods were utilized to account for pleiotropy. Finally, these methods confirmed causal evidence and the robustness of primary results.

## 4. Discussion

This study identified a causal relationship between CSF metabolites and MS by integrating evidence from MR and sensitivity analysis. Among them, the beta-alanine, 1-stearoyl-2-arachidonoyl-gpc (18:0/20:4), and allantoin were positively correlated with the risk of outcome, indicating them to be risk factors for MS. On the other hand, TMAP, butyrate (4:0) and N-acetylglycine were negatively correlated with the risk of outcome, suggesting them to be protective factors for MS.

β-Alanine is a nonessential amino acid with various physiological function, such as delaying muscle fatigue, combating acidic environments, and antioxidation. However, these effects mainly occur in muscle tissues or blood, and their specific effects in CSF still remain unclear. Previous studies^[13]^ have revealed elevated level of glycine in the CSF among MS patients, which may be a compensatory mechanism for impaired glycine transmission. In this study, higher levels of β-alanine were also confirmed in CSF of MS patients, suggesting similar perspectives.

1-Stearoyl-2-arachidonoyl-gpc is a glycerophospholipid formed from the combination of stearic acid and arachidonic acid.^[36]^ This investigation postulated that it may play an important role in the onset of MS. To be more specific, 1-stearoyl-2-arachidonoyl-gpc contains arachidonic acid (AA) at the sn-2 position and is a principal substrate for cytosolic phospholipase A2. Cytosolic phospholipase A2 preferentially cleaves AA-rich phospholipids, releasing free AA for eicosanoid biosynthesis and generating lysophosphatidylcholine, both of which are central to neuroinflammatory signaling.^[37,38]^ Therefore, both depletion (enhanced turnover) and accumulation (remodeling/compensation) of 1-stearoyl-2-arachidonoyl-gpc are biologically plausible markers of membrane remodeling and inflammatory tone. In addition, lysophosphatidylcholine species derived from PC hydrolysis can activate microglia and amplify neuroinflammation, providing a second mechanistic link between PC (18:0/20:4) turnover and immune signaling in the central nervous system.^[39,40]^

Allantoin is a natural, safe, and nontoxic imidazole heterocyclic compound, exhibiting various pharmacological effects including wound healing promotion, antitumor activation, and enhancing memory. Allantoin is a product of nonenzymatic, reactive oxygen species, which mediates oxidation of uric acid (UA) in humans and serves as an oxidative-stress readout.^[41]^ Its positive association with MS is consistent with the recognized role of oxidative injury in demyelination, mitochondrial dysfunction, and axonal damage.^[42]^ It is noteworthy that UA itself can be reduced in MS patients, so normalization to UA (allantoin/UA ratio) may better capture redox imbalance than either metabolite alone.^[43]^

There is no evidence suggesting the correlation between oxidation of UA levels in CSF and activated production of allantoin among patients with MS,^[43]^ which is inconsistent with our research and requires further investigation. Although the precise biological source of TMAP remains unknown, we propose that it could originate from the degradation of myosin light chain based on previous investigation. Nevertheless, additional research is required to verify the biological origin and possible physiological roles of TMAP.^[44]^ N-acetylglycine is a derivative of glycine, deriving from metabolic activities in various organs. In the field of medicine, the side chain of N-acetylglycine plays a crucial role in the antibacterial activity of xanthin. Moreover, a study^[45]^ showed that acetylglycine may exert anti-obesity effects on both overall and central adiposity. But this study suggested N-acetylglycine to be a protective factor for MS, which was different from our results and required further validation.

Butyrate is a molecule with versatile function. A study^[46]^ demonstrated that sodium butyrate possesses neuroprotective effects in neurological diseases. The neuroprotective impact and underlying pathways of sodium butyrate were investigated in mouse model of Parkinson disease, which was associated with colonic stimulation of GLP-1secretion. Our study also demonstrated that butyrate (4:0) was a protective factor against MS, which was consistent with previous findings. The pathogenesis of MS constitutes multifaceted process entailing interplay of multiple biological events. Changes in microenvironment, as one of the mechanisms of MS, often result in changes of CSF composition among MS patients, indicating different metabolic states and immune responses.

This MR study has some advantages. For the first time, the causal relationship between CSF metabolites and MS was demonstrated. Secondly, the all individuals are only from Europe, minimizing population stratification to the greatest extent. In addition, *F*-statistics all IVs exceed 10, providing robust statistical reliability and minimizing the influence of bias. Ultimately, several sensitivity analyses were conducted to verify the robustness of our results. Nevertheless, some limitations should be addressed. First, regardless of the sufficient power of our results, these findings should be interpreted cautiously and further validated in larger CSF-specific metabolomic GWAS data because of limited sample size. In addition, future researches should pay attention to detailed mechanisms of identified CSF metabolites and pathogenesis of MS. Furthermore, care should be taken when applying these findings to populations outside of Europe, given potential interethnic differences in genetic architecture, cerebrospinal fluid metabolites expression profiles, and disease susceptibility. The applicability of the identified associations across diverse ancestral groups remains uncertain, and future studies in multiethnic cohorts are needed to evaluate the consistency and relevance of these findings in broader populations. In addition, comprehensive pathway analysis is needed to validate our conclusions and address the current gaps.

## 5. Conclusion

In summary, we present convincing results suggesting a causal relationship between beta-alanine, 1-stearoyl-2-arachidonoyl-gpc (18:0/20:4), allantoin, TMAP, butyrate (4:0), N-acetylglycine, and MS risk. Our results indicate that interventions aimed at CSF metabolites could serve as preventive measures for MS. However, additional investigation is required to elucidate underlying biological mechanisms.

## Acknowledgments

We express our gratitude to all participants and researchers involved in the corresponding GWAS for publicly providing summary-level data.

## Author contributions

**Resources:** Jiaojiao Feng.

**Software:** Jing Feng.

**Writing – original draft:** Dejin Zhang.

**Writing – review & editing:** Gang Chen.

## Supplementary Material




